# Building a Remote Network for Sustainable Supervision in Family Medicine Residency

**DOI:** 10.1002/jgf2.70083

**Published:** 2025-11-12

**Authors:** Junki Shimokawa, Yuki Otsuka, Marina Kawaguchi, Akemi Ando, Kazushige Fujiwara

**Affiliations:** ^1^ Department of General Medicine Mine City Hospital Yamaguchi Japan; ^2^ Expert Training Support Committee of Japan Primary Care Association in the Chugoku Region Okayama Japan; ^3^ Department of General Medicine Okayama University Graduate School of Medicine, Dentistry and Pharmaceutical Sciences Okayama Japan; ^4^ Ando Occupational Health Consultant Office Tokyo Japan; ^5^ Department of International Cooperation for Medical Education, International Research Center for Medical Education Graduate School of Medicine, the University of Tokyo Tokyo Japan; ^6^ Oomagari Clinic Shimane Japan

To the Editor,

1

We are members of the Expert Training Support Committee of the Japan Primary Care Association (JPCA) in the Chugoku region and are involved in generalist education at each program. While the need for generalists is rapidly increasing, we face several challenges in supporting its education. In this context, we were especially encouraged by the recent letter from Matsumura et al. on “Sustainable Generalism Education.” [1] The Chugoku region consists of five prefectures with both mountainous and island areas, and, as shown in Figure 1, supervising doctors and family medicine residents are geographically dispersed. Against this backdrop, their call for interdisciplinary collaboration, community‐based educational guidance, and cross‐organizational sharing of best practices strongly resonates with us.

**FIGURE 1:**
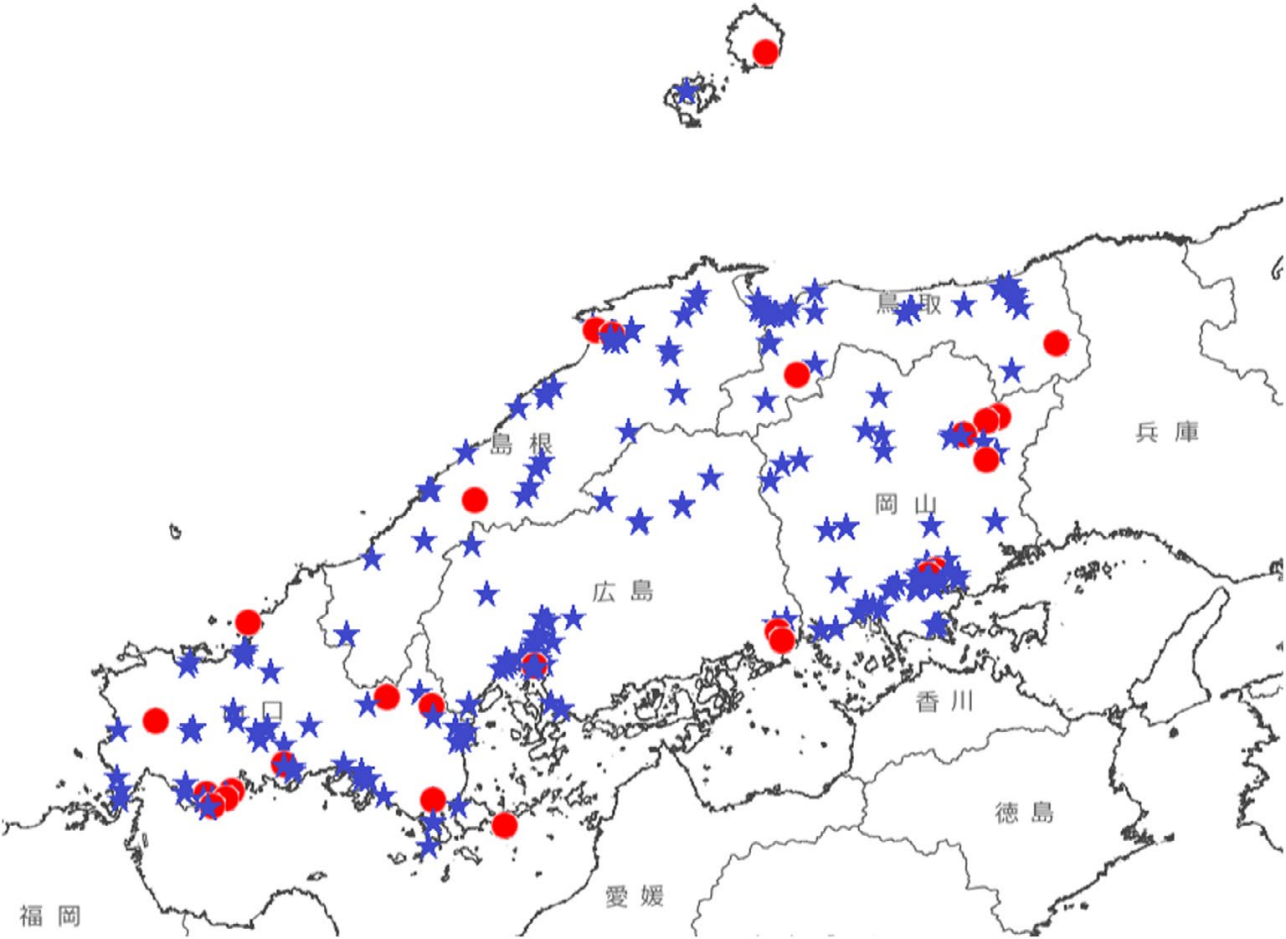
Locations of residents and supervising doctors in Chugoku region. Blue stars and red circles indicate family medicine residents and supervising doctors, respectively. The residents are dispersed, and some are not located in the same place as their supervising doctors.

What we want to emphasize is the need for support for residents who train in remote areas and for supervising doctors engaged in their education. Because generalist education in Japan is still in its early stages, the number of skillful supervising doctors is limited and tends to be concentrated at specific training sites. Meanwhile, residents are required to rotate through diverse clinical settings, and some residents are obliged by scholarship programs to work in resource‐limited rural areas. In such contexts, residents may be forced to practice independently without nearby supervisors, missing opportunities for proper reflection, while routine supervision may be provided by physicians who are inexperienced in medical education. We consider these to be major challenges in this field.

One of the greatest challenges is portfolio education. Portfolios are essential to the development of a generalist identity, but they require specialized teaching skills and sufficient time [2, 3]. Ideally, residents should be trained under a skillful supervising doctor and reflect on their practice. However, as noted above, geographical distance makes such interactions difficult. Moreover, supervising doctors at host institutions may not be familiar with portfolio education, and it is challenging to obtain a comprehensive overview of the wide range of topics involved. In particular, just‐started‐up programs often lack accumulated know‐how and tend to depend heavily on the residents' ability to learn autonomously.

In recent years, online learning opportunities in our specialty have been increasing [4, 5]. We have been discussing how these resources might help overcome current educational challenges. One of our efforts has been to open online sessions, conducted by well‐established programs, to residents and their supervising doctors in our region. Through these sessions, participants could attend core lectures and learn methods of reflection. Interestingly, most participants were not residents but supervising doctors, highlighting a strong need for learning among educators themselves. This activity also created opportunities for interaction among supervisors, enabling them to discuss shared difficulties. From this experience, we realized that in addition to supporting isolated residents, creating a peer mentoring community for supervising doctors is equally important.

We believe that building a digital network connecting supervising doctors will be key to implementing high‐quality generalist education in the future. By facilitating peer‐to‐peer learning among educators and exploring innovative educational systems, this network has the potential not only to address regional educational challenges but also to contribute to the development of a universal model of “sustainable general practice education,” as highlighted by Matsumura et al. [1].

## Author Contributions

All authors contributed to the development of the study concept. Junki Shimokawa drafted the manuscript. Yuki Otsuka revised the manuscript and created the figure. Marina Kawaguchi collected and organized the data. Akemi Ando provided guidance for the interpretation and discussion. Kazushige Fujiwara supervised the overall study. All authors reviewed and approved the final manuscript.

## Conflicts of Interest

The authors declare no conflicts of interest.

## Data Availability

The authors have nothing to report.
